# Supplementary material for: Enhanced flux potential analysis links changes in enzyme expression to metabolic flux
Source: Mol Syst Biol. 2025 Feb 17;21(4):413–45. doi: 10.1038/s44320-025-00090-9 (PMC11965317; doi:10.1038/s44320-025-00090-9)
Supplement: Supplementary file 16 — Expanded View Figures [file 44320_2025_90_MOESM16_ESM.pdf]

Expanded View Figures

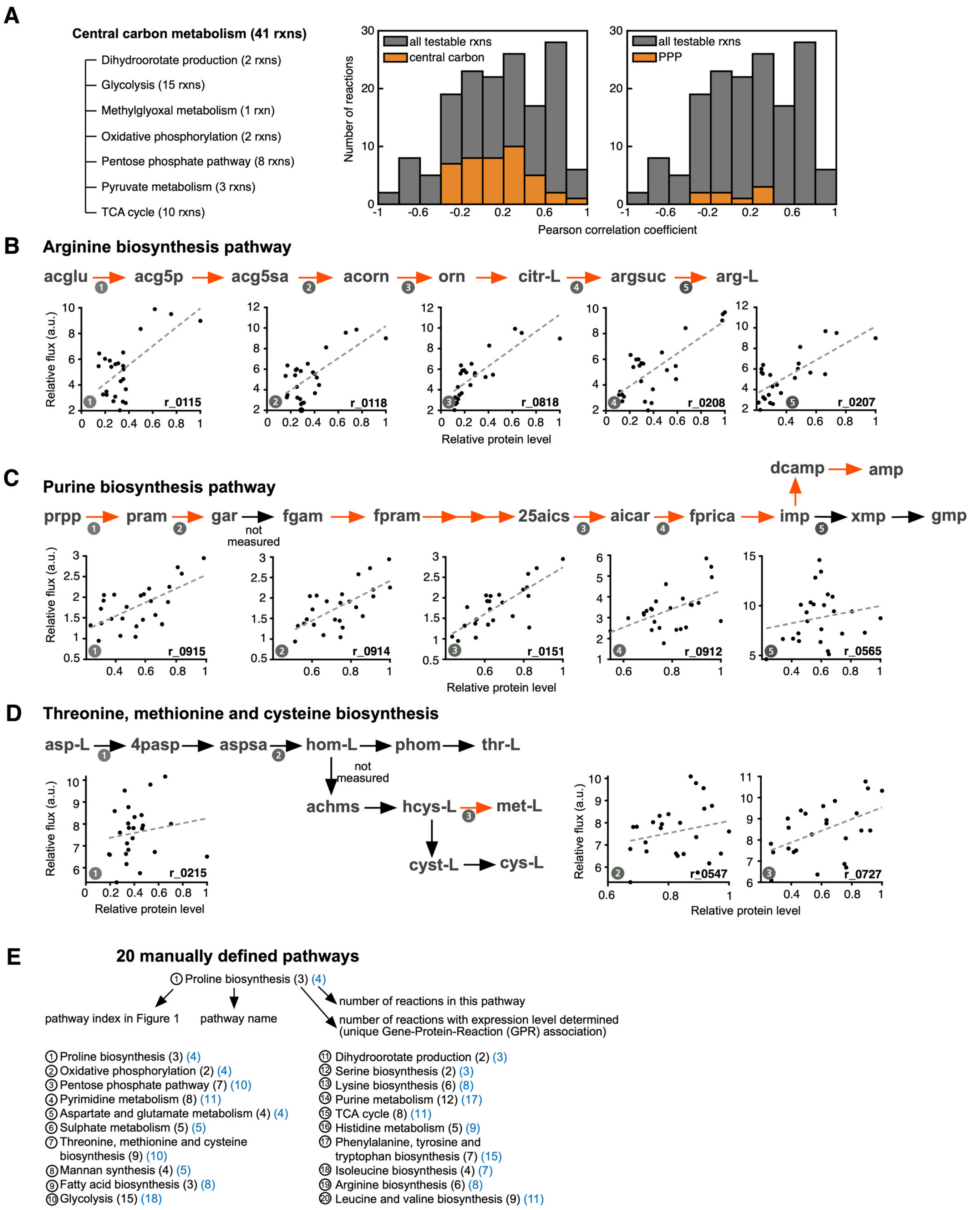

© The Author(s)

Figure EV1. Analysis of flux-enzyme level correlation in metabolic pathways.

(**A**) The PCC distribution for reactions of central carbon metabolism. The distribution of reactions of the pentose phosphate pathway (PPP) is shown on the right. (**B–D**), overlay of the flux-enzyme level correlations on selected metabolic pathways. The three selected pathways include ones that are fully (**B**) or partially (**C**) composed of correlated reactions, and one that has only one correlated reaction (**D**). Orange arrows indicate reactions that show significant correlation (FDR < 0.05, PCC > 0) and black arrows indicate uncorrelated reactions. (**E**) Twenty manually-defined pathways that are labeled by their indices in Fig. 1E, F.

© The Author(s)

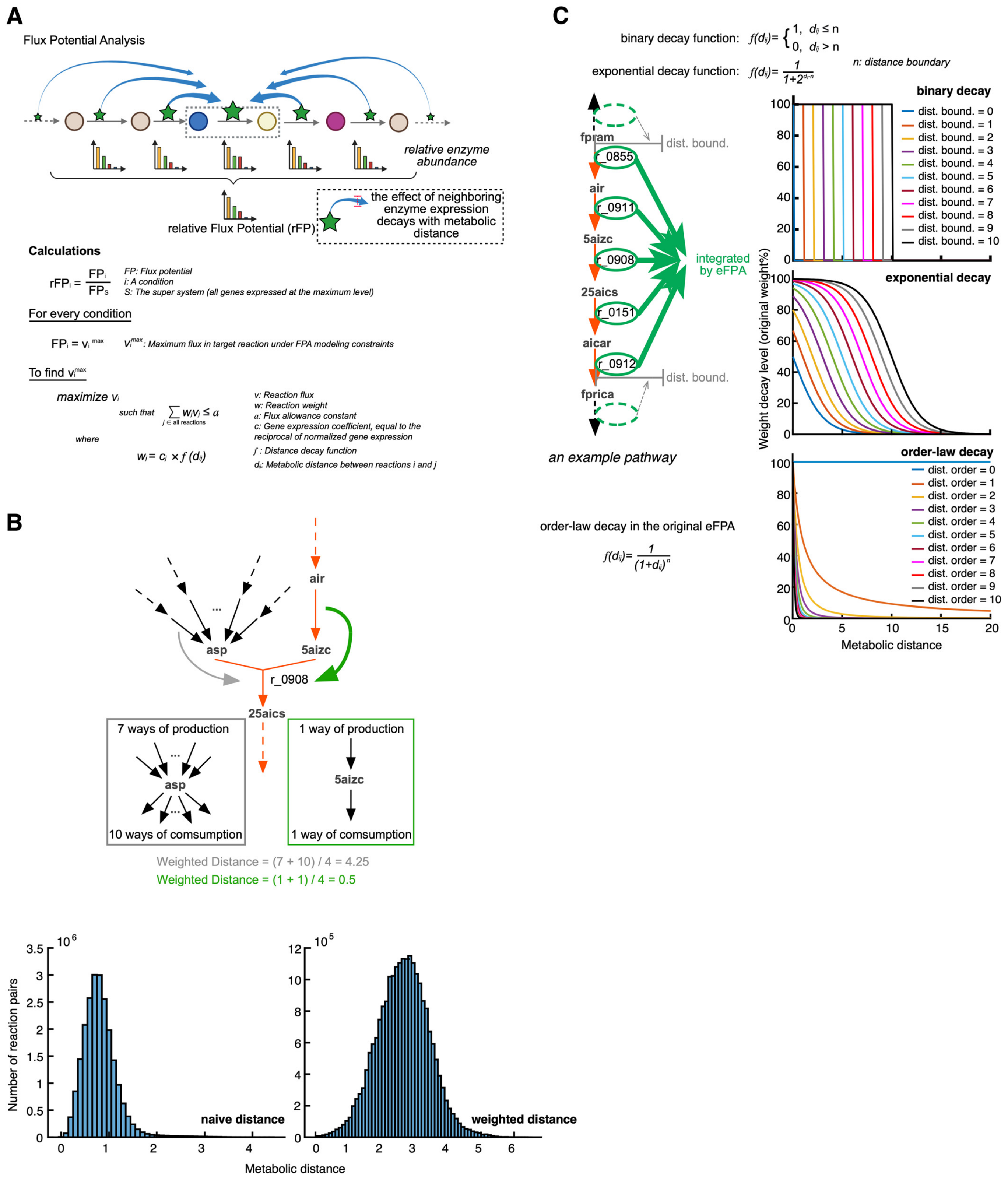

$$rFP_i = \frac{FP_i}{FP_S}$$

$$FP_i = v_i^{max}$$

v_i^{max}: Maximum flux in target reaction under FPA modeling constraints

$$\text{maximize } v_i \quad \text{such that} \sum_{j \in \text{all reactions}} w_j v_j \le a$$

$$w_j = c_j \times f(d_{ij})$$

$$f(d_{ij}) = \begin{cases} 1, & d_{ij} \le n \\ 0, & d_{ij} > n \end{cases}$$

$$f(d_{ij}) = \frac{1}{1+2^{d_{ij}-n}}$$

$$f(d_{ij}) = \frac{1}{(1+d_{ij})^n}$$

 © The Author(s)

◀ **Figure EV2. Principles of FPA and eFPA.**

(**A**) Schematic of flux potential analysis (FPA). FPA and eFPA integrate the levels of enzymes that catalyze reactions surrounding a ROI to predict relative flux potential (rFP) of the ROI. The contribution of surrounding enzymes can be tuned by a distance decay function such that FPA is versatile to integrate expression information from a local subnetwork to the entire network. The mathematical formulation of FPA is briefly summarized (also see Appendix Text S2). (**B**) The weighted metabolic distance. A cartoon illustrating the calculation of weighted metabolic distance is shown on the top. On the bottom, the distributions of naïve (unweighted) and weighted metabolic distance are shown for all reaction pairs in the yeast metabolic model. (**C**) The distance decay functions of eFPA. The formulas of binary (used in optimal-boundary eFPA) and exponential decay functions (used in standard eFPA) are shown in the figure. The left panel shows a cartoon to illustrate the concept of distance boundary. The right panel shows the decay curve of the two functions.

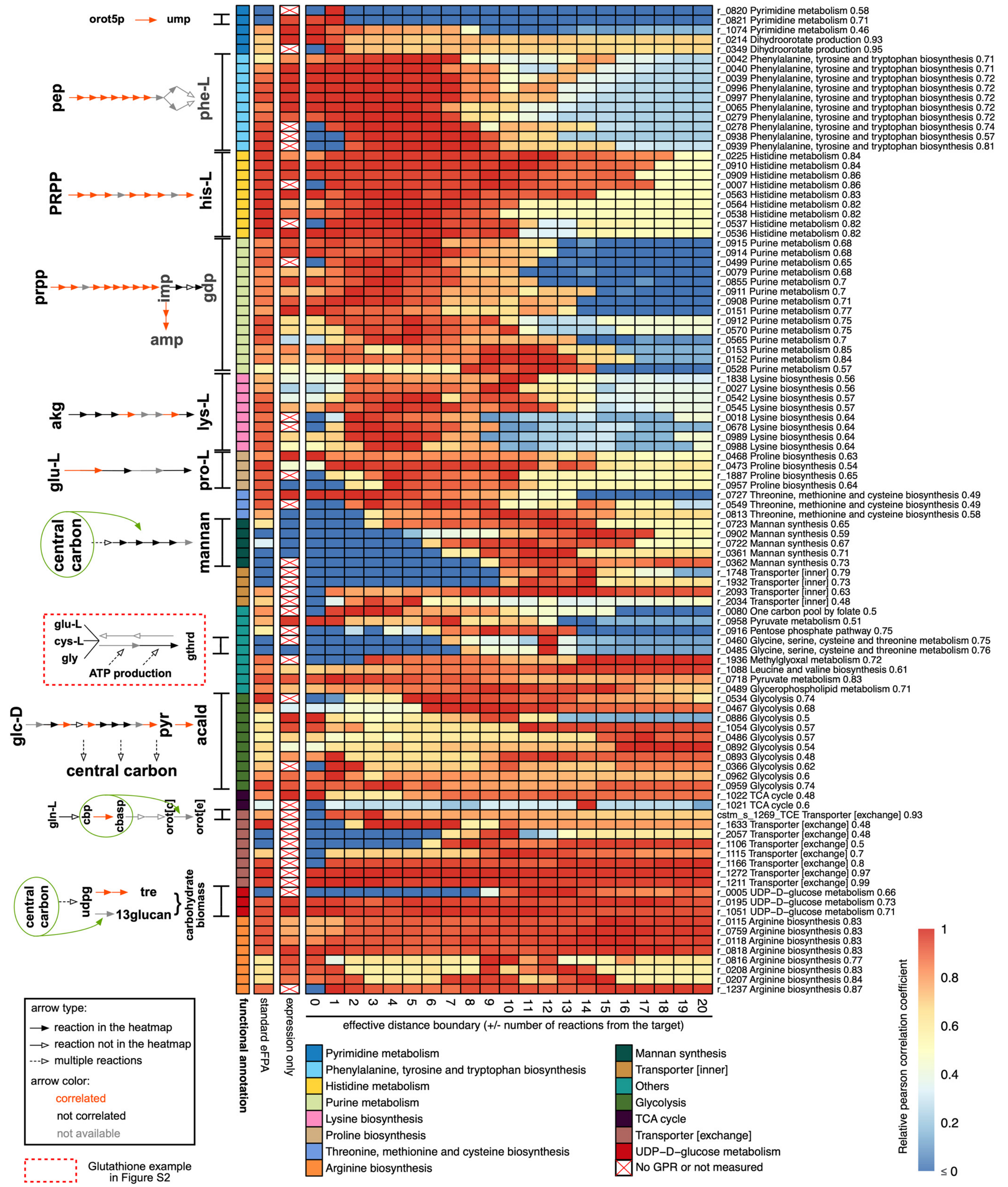
orot5p
ump
pep
phe-L
PRPP
his-L
prpp
imp
gdp
amp
akg
lys-L
glu-L
pro-L
central carbon
mannan
glu-L
cys-L
gly
ATP production
gthrd
glc-D
pyr
acald
central carbon
gln-L
cbp
cbasp
orot[c]
orot[e]
central carbon
udpg
tre
13glucan
carbohydrate biomass
r_0820 Pyrimidine metabolism 0.58
r_0821 Pyrimidine metabolism 0.71
r_1074 Pyrimidine metabolism 0.46
r_0214 Dihydroorotate production 0.93
r_0349 Dihydroorotate production 0.95
r_0042 Phenylalanine, tyrosine and tryptophan biosynthesis 0.71
r_0040 Phenylalanine, tyrosine and tryptophan biosynthesis 0.71
r_0039 Phenylalanine, tyrosine and tryptophan biosynthesis 0.72
r_0996 Phenylalanine, tyrosine and tryptophan biosynthesis 0.72
r_0997 Phenylalanine, tyrosine and tryptophan biosynthesis 0.72
r_0065 Phenylalanine, tyrosine and tryptophan biosynthesis 0.72
r_0279 Phenylalanine, tyrosine and tryptophan biosynthesis 0.72
r_0278 Phenylalanine, tyrosine and tryptophan biosynthesis 0.74
r_0938 Phenylalanine, tyrosine and tryptophan biosynthesis 0.57
r_0939 Phenylalanine, tyrosine and tryptophan biosynthesis 0.81
r_0225 Histidine metabolism 0.84
r_0910 Histidine metabolism 0.84
r_0909 Histidine metabolism 0.86
r_0007 Histidine metabolism 0.86
r_0563 Histidine metabolism 0.83
r_0564 Histidine metabolism 0.82
r_0538 Histidine metabolism 0.82
r_0537 Histidine metabolism 0.82
r_0536 Histidine metabolism 0.82
r_0915 Purine metabolism 0.68
r_0914 Purine metabolism 0.68
r_0499 Purine metabolism 0.65
r_0079 Purine metabolism 0.68
r_0855 Purine metabolism 0.7
r_0911 Purine metabolism 0.7
r_0908 Purine metabolism 0.71
r_0151 Purine metabolism 0.77
r_0912 Purine metabolism 0.75
r_0570 Purine metabolism 0.75
r_0565 Purine metabolism 0.7
r_0153 Purine metabolism 0.85
r_0152 Purine metabolism 0.84
r_0528 Purine metabolism 0.57
r_1838 Lysine biosynthesis 0.56
r_0027 Lysine biosynthesis 0.56
r_0542 Lysine biosynthesis 0.57
r_0545 Lysine biosynthesis 0.57
r_0018 Lysine biosynthesis 0.64
r_0678 Lysine biosynthesis 0.64
r_0989 Lysine biosynthesis 0.64
r_0988 Lysine biosynthesis 0.64
r_0468 Proline biosynthesis 0.63
r_0473 Proline biosynthesis 0.54
r_1887 Proline biosynthesis 0.65
r_0957 Proline biosynthesis 0.64
r_0727 Threonine, methionine and cysteine biosynthesis 0.49
r_0549 Threonine, methionine and cysteine biosynthesis 0.49
r_0813 Threonine, methionine and cysteine biosynthesis 0.58
r_0723 Mannan synthesis 0.65
r_0902 Mannan synthesis 0.59
r_0722 Mannan synthesis 0.67
r_0361 Mannan synthesis 0.71
r_0362 Mannan synthesis 0.73
r_1748 Transporter [inner] 0.79
r_1932 Transporter [inner] 0.73
r_2093 Transporter [inner] 0.63
r_2034 Transporter [inner] 0.48
r_0080 One carbon pool by folate 0.5
r_0958 Pyruvate metabolism 0.51
r_0916 Pentose phosphate pathway 0.75
r_0460 Glycine, serine, cysteine and threonine metabolism 0.75
r_0485 Glycine, serine, cysteine and threonine metabolism 0.76
r_1936 Methylglyoxal metabolism 0.72
r_1088 Leucine and valine biosynthesis 0.61
r_0718 Pyruvate metabolism 0.83
r_0489 Glycerophospholipid metabolism 0.71
r_0534 Glycolysis 0.74
r_0467 Glycolysis 0.68
r_0886 Glycolysis 0.5
r_1054 Glycolysis 0.57
r_0486 Glycolysis 0.57
r_0892 Glycolysis 0.54
r_0893 Glycolysis 0.48
r_0366 Glycolysis 0.62
r_0962 Glycolysis 0.6
r_0959 Glycolysis 0.74
r_1022 TCA cycle 0.48
r_1021 TCA cycle 0.6
cstm_s_1269_TCE Transporter [exchange] 0.93
r_1633 Transporter [exchange] 0.48
r_2057 Transporter [exchange] 0.48
r_1106 Transporter [exchange] 0.5
r_1115 Transporter [exchange] 0.7
r_1166 Transporter [exchange] 0.8
r_1272 Transporter [exchange] 0.97
r_1211 Transporter [exchange] 0.99
r_0005 UDP–D–glucose metabolism 0.66
r_0195 UDP–D–glucose metabolism 0.73
r_1051 UDP–D–glucose metabolism 0.71
r_0115 Arginine biosynthesis 0.83
r_0759 Arginine biosynthesis 0.83
r_0118 Arginine biosynthesis 0.83
r_0818 Arginine biosynthesis 0.83
r_0816 Arginine biosynthesis 0.77
r_0208 Arginine biosynthesis 0.83
r_0207 Arginine biosynthesis 0.84
r_1237 Arginine biosynthesis 0.87
functional annotation
standard eFPA
expression only
0 1 2 3 4 5 6 7 8 9 10 11 12 13 14 15 16 17 18 19 20
effective distance boundary (+/- number of reactions from the target)
arrow type:
reaction in the heatmap
reaction not in the heatmap
multiple reactions
arrow color:
correlated
not correlated
not available
Glutathione example in Figure S2
Pyrimidine metabolism
Phenylalanine, tyrosine and tryptophan biosynthesis
Histidine metabolism
Purine metabolism
Lysine biosynthesis
Proline biosynthesis
Threonine, methionine and cysteine biosynthesis
Arginine biosynthesis
Mannan synthesis
Transporter [inner]
Others
Glycolysis
TCA cycle
Transporter [exchange]
UDP–D–glucose metabolism
No GPR or not measured
Relative pearson correlation coefficient
1
0.8
0.6
0.4
0.2
≤ 0

Figure EV3. Heatmap of PCC between flux and rFP with a titration of distance boundary.

The relative PCC (row-wise normalized by dividing each value by the row maximum) for 102 predicted reactions. The numbers on x-axis indicate the effective distance boundary, which represents the calculated actual length of the integrated pathway based on the distance boundary parameter in the scale of weighted metabolic distance. ROIs (rows) are arranged based on their position in the pathway they are associated with, and reaction IDs are indicated on y-axis together with the associated pathway and the optimal-boundary PCC. Significant contributors (e.g., relevant pathway reactions) to eFPA analysis of some ROIs are depicted on the left.

© The Author(s)

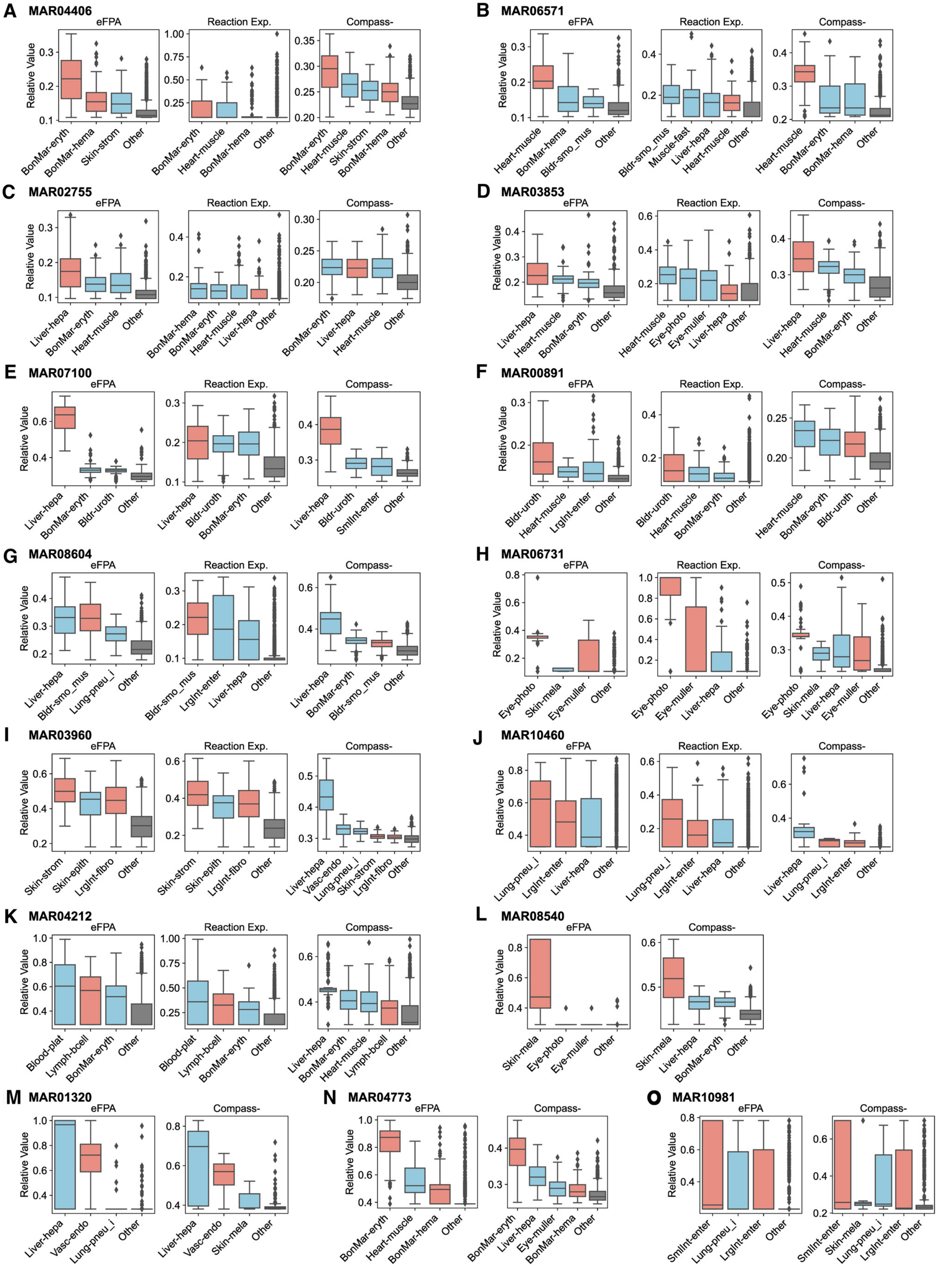
A MAR04406
eFPA
Reaction Exp.
Compass-
Relative Value
BonMar-eryth
BonMar-hema
Skin-strom
Other
Heart-muscle
B MAR06571
Bldr-smo_mus
Muscle-fast
Liver-hepa
C MAR02755
D MAR03853
Eye-photo
Eye-muller
E MAR07100
Bldr-uroth
SmInt-enter
F MAR00891
LrgInt-enter
G MAR08604
Lung-pneu_i
H MAR06731
Skin-mela
I MAR03960
Skin-epith
LrgInt-fibro
Vasc-endo
J MAR10460
K MAR04212
Blood-plat
Lymph-bcell
L MAR08540
M MAR01320
N MAR04773
O MAR10981

© The Author(s)

◀ **Figure EV4. Verifiable cell-type-enriched cytosolic reactions in Human 1.**

Examples in Table 1 are displayed as box plots, with cell types of interest highlighted in salmon. In addition to these highlighted cell types, the top three cell types based on the predicted flux activity are also shown in each plot, unless they overlap with the highlighted cell types. (**A–K**) Examples where reaction expression or Compass- fails to clearly differentiate one or more of the referred cell types of interest from Table 1. (**A**) The activity of a primary purine biosynthesis reaction is not well-differentiated by reaction expression in erythroid progenitors and is missed by both reaction expression and Compass- in hematopoietic stem cells. (**B–D**), Activities of indicated reactions are missed by reaction expression in the cell type of interest (Table 1). (**E**) Xenobiotic detoxification reaction activity in hepatocytes is poorly differentiated by reaction expression. (**F**, **G**, **I–K**) Activities of indicated reactions are missed by Compass- in the cell type(s) of interest (Table 1). (**H**) Dopamine production potential in Muller cells is comparatively low in Compass- evaluations, especially against hepatocytes and melanocytes. (**L–O**) Activities of reactions without gene associations, predicted only by eFPA and Compass-. While both methods generally provide accurate cell-type enrichments, eFPA typically offers better differentiation of the expected cell types (Table 1). All box plots display the median (central line), IQR (box boundaries), and whiskers extending to the nearest data points within 1.5*IQR from the first and third quartiles. Points outside this range, when present, are depicted as outliers. Distributions for each cell type are illustrated with the number of data points (*n*) per cell type as specified in the table in Fig. 5A. The "Other" category includes all remaining data points not covered by the listed cell types. See Fig. 5B for a detailed example.

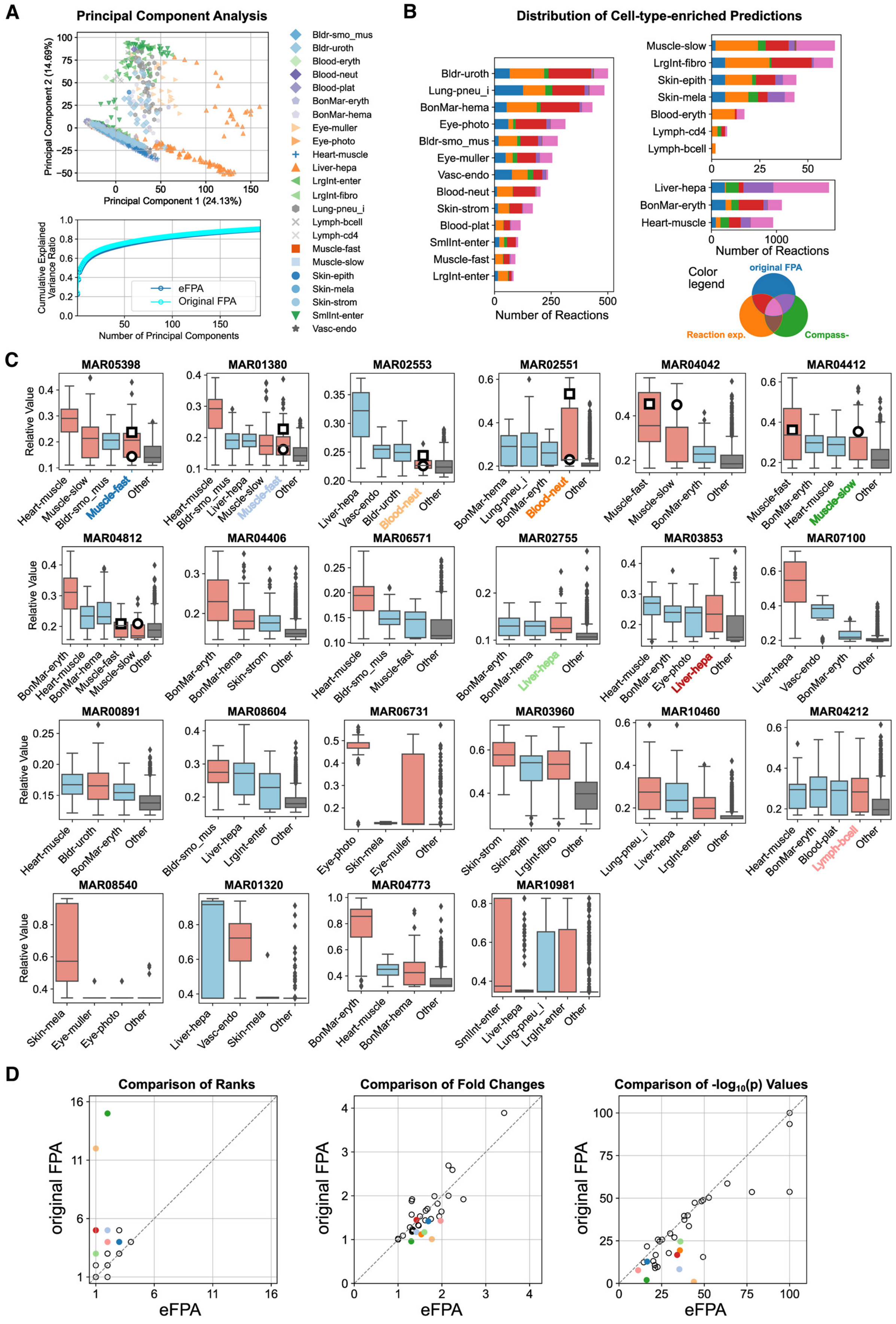
A
Principal Component Analysis
Principal Component 2 (14.69%)
Principal Component 1 (24.13%)
Bldr-smo_mus
Bldr-uroth
Blood-eryth
Blood-neut
Blood-plat
BonMar-eryth
BonMar-hema
Eye-muller
Eye-photo
Heart-muscle
Liver-hepa
LrgInt-enter
LrgInt-fibro
Lung-pneu_i
Lymph-bcell
Lymph-cd4
Muscle-fast
Muscle-slow
Skin-epith
Skin-mela
Skin-strom
SmlInt-enter
Vasc-endo
Cumulative Explained Variance Ratio
eFPA
Original FPA
Number of Principal Components
B
Distribution of Cell-type-enriched Predictions
Number of Reactions
Color legend
original FPA
Reaction exp.
Compass-
C
Relative Value
MAR05398
MAR01380
MAR02553
MAR02551
MAR04042
MAR04412
MAR04812
MAR04406
MAR06571
MAR02755
MAR03853
MAR07100
MAR00891
MAR08604
MAR06731
MAR03960
MAR10460
MAR04212
MAR08540
MAR01320
MAR04773
MAR10981
Other
D
Comparison of Ranks
Comparison of Fold Changes
Comparison of -log10(p) Values
original FPA
eFPA

Figure EV5. Integration of single-cell data with original FPA to enable comparison with eFPA results.

(**A**) PCA of predicted relative reaction flux potentials. Top panel shows Clustering of single cells using first two principal components (as in Fig. 5B); bottom panel shows cumulative variance explained by principal components (as in Fig. 5C). Percent variance explained is indicated on the axes (top panel). (**B**) Distribution of cell-type-enriched reactions for each cell type (as in Fig. 5E). (**C**) Box plots of FPA predictions (as in Figs. 6B, D, G, I and EV4), with cell types of interest highlighted in salmon. Colored cell types in x-axis labels match colored data points highlighted in (**D**). The plots display the median (central line), IQR (box boundaries), and whiskers extending to the nearest data points within 1.5*IQR from the first and third quartiles. Points outside this range, when present, are depicted as outliers. Distributions for each cell type are illustrated with the number of data points (*n*) per cell type as specified in the table in Fig. 5A. The "Other" category includes all remaining data points not covered by the listed cell types. See Fig. 5D for a detailed example. (**D**) Comparison of predictive power metrics (rank, fold change, and *p*-values; Table 1, Table EV2) between eFPA and FPA. Colored data points indicate predictions where eFPA significantly outperforms FPA, with colors matching those in (**C**). In the log-transformed *p*-value plot, values above 100 are capped at 100 for visualization.

© The Author(s)
